# Application of Boroisoquinoline Fluorophores as Chemodosimeters for Fluoride Ion and Pd (0)

**DOI:** 10.3390/ma13010199

**Published:** 2020-01-02

**Authors:** Dénes Sóvári, György Miklós Keserű, Péter Ábrányi-Balogh

**Affiliations:** Research Centre for Natural Sciences, Institute of Organic Chemistry, Medicinal Chemistry Research Group, POB 286, 1519 Budapest, Hungary; sovari.denes@ttk.hu (D.S.); keseru.gyorgy@ttk.hu (G.M.K.)

**Keywords:** boroisoquinolines, chemodosimeter, fluorescence, fluorescent molecular probe, fluoride, palladium (0)

## Abstract

The development of novel chemodosimeters is currently a prosperous field in organic chemistry. Recently, a new family of fluorophores, the boroisoquinolines, were introduced with satisfying photophysical properties. As a continuation of this research, the application of boroisoquinolines is presented as chemodosimeters for fluoride anion and Pd (0). The new tools showed good selectivity for the detection of the analytes. Moreover, the mechanism of action was investigated experimentally.

## 1. Introduction

Chemodosimeters and chemosensors are handy chemical tools for the detection of ions, metals, or reactive species. In most cases, it is prominently significant that the analyte has biological activity or at least some effect on living organisms [1,2]. The mechanism of action of the chemodosimeters is usually based on an irreversible chemical reaction with the analyte that changes the structure of the probe, and causes a well-sensible shift in fluorescence properties. This might be an on/off signal (turn-on and turn-off probes) [3]. The first fluoride chemodosimeter was published in 2000 [4,5], while the first compound that was able to detect Pd (0) besides Pd^2+^ was reported in 2007 [6]. Notably, since then, the number of fluorescent molecular probes have been rising rapidly [7] due to the needs of fast and on-the-spot analytical testing that turned inevitable the development of this frontier field of organic and analytical chemistry.

**Figure 1:**
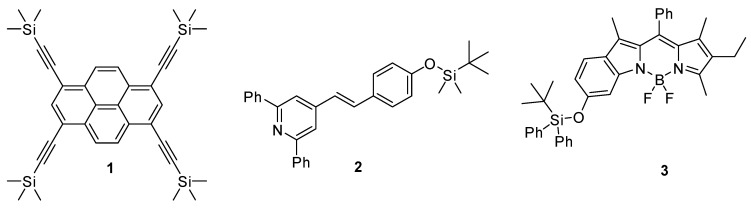
Irreversible fluoride-selective fluorescent probes.

Although fluoride anions are elemental for bone and tooth health, it was recognized that the presence of fluoride in a larger amount might cause skeletal fluorosis, calcification, and weakening of the bone tissue [8,9,10]. Thus, in rural areas of developing countries, where one of the main water-polluting component is the fluoride anion [1], it became important to find analytical tools for its recognition. This induced the development of various fluoride fluorescent probes: reversible and irreversible ones (**1**–**3**) as well. The mechanism of the reversible compounds rests on salt formation [11,12], while the irreversibly acting substances contain generally a C–Si (**1**) or O–Si (**2,3**) bond as a recognition site (Figure 1), and take advantage of the high reactivity of fluoride towards silicon [13,14,15,16]. There are five other types of fluorescens fluoride test compounds: organoboron compounds, nucleophilic addition based probes, intramolecular hydrogen transfer reaction based agents, metal-mediated probes, and bifunctional fluorescent test compounds [1].

**Figure 2:**
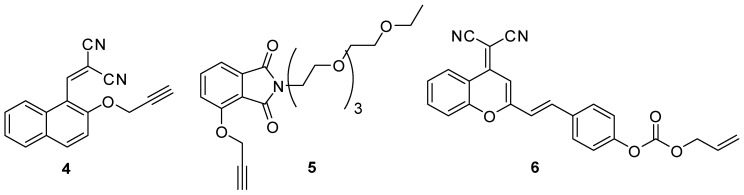
Palladium chemodosimeters.

Palladium is widely used in transition metal-catalyzed reactions and for reductions in chemical research and industry as well as in the catalysators of exhaust-pipes in vehicles [17]. The emerging number of cars with palladium containing catalyst results in the raise of the palladium-level in the air causing allergic symptoms and other maleficent biological effects when getting into living organisms [2]. The Pd (0) chemodosimeters are usually based on catalytic processes. In most cases, the fluorophore skeleton is equipped with an *O*-allyl or *O*-propargyl group, which is removed by the palladium provoking the sensible photophysical change [17,18,19]. The photospectroscopic properties might change due to a ring closure by intramolecular acylation (**4**), or the by the removal of the *O*-propargyl (**5**) or the *O*-(*O*-allyl) carboxyl group (**6**), which turns on the ESIPT (Excited State Intramolecular Proton Transfer) effect (Figure 2).

**Figure 3:**
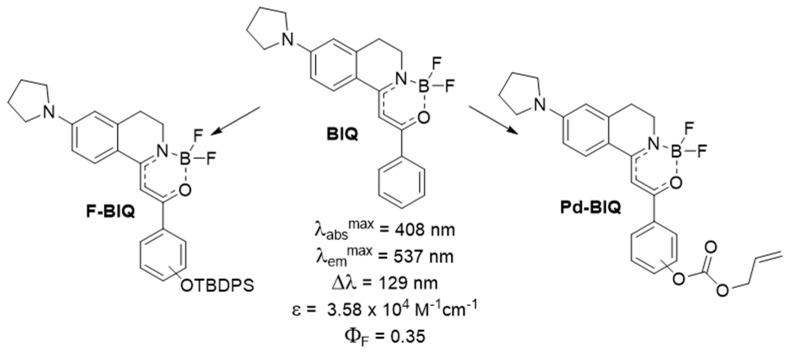
Member of the boroisoquinoline family (**BIQ**) and the proposed chemodosimeters (**F-BIQ**, **Pd-BIQ**).

Recently, we developed a new fluorescent compound family, namely the boroisoquinolines (Figure 3, **BIQ**), showing good photophysical properties [20]. As the continuation of our research, we aimed to synthetize derivatives for analytical applications, in particular for the detection of fluoride (**F-BIQ**) and palladium (**Pd-BIQ**), as a development of chemodosimeters based on the novel fluorophores (Figure 3). We envisaged that the recognition elements would be the *tert*-butyldiphenylsilyl group for the fluoride anion, and the *O*-allyl moiety for Pd (0) sensing.

## 2. Materials and Methods

All melting points were determined on a Jasco SRS OptiMelt apparatus (Stanford, CA, USA) and are uncorrected. ^1^H and ^13^C NMR spectra were recorded in CDCl_3_ or DMSO-*d*_6_ solution at room temperature, on a Varian Unity Inova 500 spectrometer (500 and 125 MHz for ^1^H and APT NMR spectra, respectively), and on a Varian Unity Inova 300 spectrometer (300, 75 MHz and 282 MHz for ^1^H, APT NMR and ^19^F NMR spectra, respectively), with the deuterium signal of the solvent as the lock and TMS as the internal standard. Chemical shifts (*δ*) and coupling constants (*J*) are given in ppm and Hz, respectively. High resolution mass spectra were recorded on a Waters Q-TOF Premier mass spectrometer in positive ESI ionization mode. The reactions were followed by analytical thin layer chromatography on silica gel 60 F_254_ and HPLC–MS chromatography with a Shimadzu LCMS-2020 device using a Reprospher 100 C18 (5 μm; 100 × 3 mm) column and positive-negative double ion source (DUIS) with a quadrupole MS analysator in a range of 50–1000 *m*/*z*. All reagents were purchased from commercial sources [e.g., Sigma Aldrich (St. Louis, MO, USA), Fluorochem (Hadfield, Derbyshire, UK), Alfa (Haverhill, MA, USA), Combi-Blocks (San Diego, CA, USA)]. Analytical samples of new compounds were obtained by trituration or recrystallization from the solvents or solvent mixtures given below in parentheses.

### 2.1. Photophysical Measurements

The fluorescence and absorbance measurements were carried out on a Jasco FP8300 spectrofluorometer, in a standard cell quartz cuvette with 1 cm light path length. The widths of the excitation slit and the emission slit were both set to 2.5 nm with the scanning speed at 1000 nm/min. Pure solvents were used as blank correction. We used acetonitrile as solvent if not otherwise mentioned, and the concentration was 1 μM. For water tests, we used 10 μM solutions of the chemodosimeters, made with pH = 7.4 PBS (phosphate buffered saline) buffer with 0.1% SDS (sodium dodecyl sulfate). We measured the absorbance spectra from 250 nm to 500 nm. The excitement spectra were recorded between 250 nm and 500 nm. For the emission spectra, we excited the sample at the excitation maximum, and recorded it from excitation maximum plus 10 nm to 800 nm. The test solutions were made in PBS puffer (50 mM phosphate, pH = 7.4) for aqueous measurements, or in acetonitrile. For the investigation of the **12a** chemodosimeter, we used tetrabutylammonium (TBA) salts, and, for the **12d** chemodosimeter, we used chloride salts and *tri* (dibenzylideneacetone) dipalladium (0) (Pd_2_dba_3_).

### 2.2. Synthetic Procedures

#### 2.2.1. General Procedure for the Preparation of 1-Methylidene-3,4-Dihydroisoquinolines (**11**)

In a three-necked round bottom flask 20 mL of anhydrous tetrahydrofuran and 0.42 mL of diisopropylamine (0.30 g, 3.0 mmol, 1.2 equiv.) were cooled below –70 °C, in a nitrogen atmosphere. Then, 3 mmol (1.2 equiv.) *n*-BuLi was added dropwise, and the reaction mixture was stirred for 1 h. Then, 2.5 mmol isoquinoline (**9**) in tetrahydrofuran solution (0.54 g) was added dropwise. The reaction mixture was stirred for 1 h, then the appropriate ester (3.0 mmol, 1.2 equiv.) was added dropwise in 10 mL tetrahydrofuran [**11a**: ethyl 4-((*tert*-butyldiphenylsilyl) oxy) benzoate (**10a**) (2.5 g, 2.5 equiv.); **11b** ethyl−4-methoxybenzoate (**10b**) (1.1 g, 2.5 equiv.)]. Then, the reaction mixture was allowed to warm to room temperature and stirred for 1 h. The solution was quenched with 30 mL water, and extracted with 80 mL ethyl acetate. The aqueous layer was washed with ethyl acetate (2 × 30 mL) and dichloromethane (2 × 30 mL). The combined organic phases were dried (MgSO_4_), filtered and evaporated under reduced pressure. The residue was purified by flash column chromatography on aluminum oxide using hexane/ethyl acetate.

*(Z)-1-(4-((tert-butyldiphenylsilyl) oxy) phenyl)-2-(6-(pyrrolidin-1-yl)-3,4-dihydroisoquinolin-1(2H)-ylidene) ethanone (**11a**)*. Yield: 2.1 g (85%), yellow crystals, m.*p*. 66–68 °C (CDCl_3_); ^1^ H NMR (300 MHz, CDCl_3_) *δ* 11.68 (s, 1H, NH), 7.75–7.73 (m, 2H, ArH), 7.73–7.71 (m, 4H, ArH), 7.62 (d, *J* = 8.8 Hz, 1H, ArH), 7.45–7.40 (m, 2H, ArH), 7.37 (t, *J* = 7.3 Hz, 4H, ArH), 6.79 (d, *J* = 8.6 Hz, 2H, ArH), 6.44 (dd, *J* = 8.8, 2.4 Hz, 1H, ArH), 6.30 (d, *J* = 2.0 Hz, 1H, ArH), 6.12 (s, 1H, HC=), 3.49 (t, *J* = 5.7 Hz, 2H, CH_2_), 3.33 (t, *J* = 6.5 Hz, 4H, CH_2_), 2.86 (t, *J* = 6.5 Hz, 2H, CH_2_), 2.05–1.99 (m, 4H, CH_2_), 1.12 (s, 9H, CH_3_) ppm; ^13^H NMR (125 MHz, CDCl_3_) *δ* 186.6 (C = O), 159.4 (C=), 157.4 (C=), 149.7 (C=), 138.3 (C=), 135.5 (=CH), 134.7 (C=), 132.8 (C=), 129.9 (=CH), 128.3 (=CH), 127.8 (=CH), 127.3 (=CH), 119.2 (=CH), 116.2 (C=), 110.21 (=CH), 84.9 (=CH), 47.5 (CH_2_), 38.8 (CH_2_), 29.1 (CH_2_), 26.5 (CH_3_), 25.4 (CH_2_), 19.5 (C=) ppm; [M + H]^+^_measured_ = 573.2917, calcd. for C_37_H_41_N_2_O_2_Si: 573.2931.

*(Z)-1-(4-methoxyphenyl)-2-(6-(pyrrolidin-1-yl)-3,4-dihydroisoquinolin-1(2H)-ylidene) ethanone (**11b**)*. Yield: 0,43 g (40%), yellow crystals, m.*p*. 186 °C (hexane-ethyl acetate); ^1^H NMR (500 MHz, CDCl_3_) *δ* 11.72 (s, 1H, NH), 7.96–7,87 (m, 2H, ArH), 7,67 (d, *J* = 8,8 Hz, 1H, ArH), 6,93–6,89 (m, 2H, ArH), 6,46 (dd, *J* = 8,7, 2,4 Hz, 1H, ArH), 6,31 (d, *J* = 2,2 Hz, 1H, ArH), 6,19 (s, 1H, HC=), 3,84 (s, 3H, OCH_3_), 3,50 (tt, *J* = 8,6, 4,3 Hz, 2H, CH_2_), 3,33 (t, *J* = 6,6 Hz, 4H, CH_2_), 2,88 (t, *J* = 6,6 Hz, 2H, CH_2_), 2,07 − 1,97 (m, 4H, CH_2_) ppm; ^13^C NMR (125 MHz, CDCl_3_) *δ* 186,5 (C = O), 160,4 (C = O), 153,5 (C=), 152,6 (C=), 150,1 (CH=), 139,9 (C=), 138,7 (CH=), 131,4 (C=), 128,4 (CH=), 127,7 (=CH), 120,8 (=CH), 119,8 (C=), 116,0 (C=), 110,6 (=CH), 110,5 (=CH), 85,5 (=CH_2_), 69,4 (CH_2_), 47,8 (CH_2_), 39,1 (CH_2_), 29,2 (CH_2_), 25,7 (CH_2_) ppm; [M + H]^+^_measured_ = 349.1908, calcd. for C_22_H_25_N_2_O_2_: 349.1910.

*(Z)-allyl (4-(2-(6-(pyrrolidin-1-yl)-3,4-dihydroisoquinolin-1(2H)-ylidene) acetyl) phenyl) carbonate (**11d**).*

In a round bottom flask, 0.87 g (*Z*)-1-(4-methoxyphenyl)-2-(6-(pyrrolidin-1-yl)-3, 4-dihydroisoquinolin-1 (2*H*)-ylidene) ethanone (**11b**) (2.5 mmol) was dissolved in 25 mL anhydrous dichloromethane. The flask was flushed with argon and cooled to 0 °C, then 7.7 mL 1 M BBr_3_ dichloromethane solution (7.5 mmol, 3 equiv.) was added dropwise. The reaction mixture was stirred at room temperature overnight. The next day it was quenched with 20 mL water; then, the water layer was extracted with dichloromethane ten times. The combined organic layers were dried (MgSO_4_), filtered, and concentrated in vacuo. Then, the residue was dissolved in 60 mL anhydrous tetrahydrofuran and 50 mL dichloromethane, 0.49 mL triethylamine (0.35 g, 3.5 mmol, 1.4 equiv.) was added and the mixture was cooled below −10 °C. In addition, 0.32 mL allyl chloroformate (0.36 g, 3 mmol, 1.2 equiv.) was added dropwise; then, the reaction mixture was let to warm room temperature and stirred overnight. The next day the reaction mixture was reduced in vacuo. The residue was purified by flash chromatography on silica gel with hexane/ethyl acetate.

Yield: 0.16 g (15%), brown film; ^1^H NMR (300 MHz, CDCl_3_) *δ* 11.79 (s, 1H, NH), 7.95 (d, *J* = 8.3 Hz, 2H, ArH), 7.66 (d, *J* = 8.7 Hz, 1H, ArH), 7.22 (d, *J* = 8.3 Hz, 2H, ArH), 6.47 (d, *J* = 8.6 Hz, 1H, ArH), 6.32 (s, 1H, ArH), 6.17 (s, 1H, HC=), 6.01 (dq, *J* = 10.6, 5.7 Hz, 1H, HC=), 5.43 (d, *J* = 17.1 Hz, 1H, HC=), 5.33 (d, *J* = 10.4 Hz, 1H, HC=), 4.75 (d, *J* = 5.7 Hz, 2H, CH_2_), 3.52 (s, 2H, CH_2_), 3.35 (s, 4H, CH_2_), 2.89 (t, *J* = 6.2 Hz, 2H, CH_2_), 2.03 (s, 4H, CH_2_) ppm; ^13^C NMR (75 MHz, CDCl_3_) *δ* 186.0 (C=O), 160.4 (C = O), 153.5 (C=), 152.6 (CH=), 150.1 (CH=), 139.6 (CH=), 138.7 (CH=), 131.4 (C=), 128.4 (CH=), 127.7 (C=), 120.8 (CH=), 119.8 (C=), 116.0 (C=), 110.6 (C=), 110.5 (CH=), 85,5 (CH_2_), 69.4 (CH_2_), 47.8 (CH_2_), 39.1 (CH_2_), 29.2 (CH_2_), 25.7 (CH_2_) ppm; [M + H]^+^_measured_ = 419.1984, calcd. for C_25_H_27_N_2_O_4_: 419.1970.

#### 2.2.2. General Procedure for the Preparation of Boroisoquinolines (**12**)

Into a round bottom flask, 1 mmol appropriate 1-methylidene-3,4-dihydroisoquinoline (**14**) was measured {(*Z*)-1-(4-((tert-butyldiphenylsilyl) oxy) phenyl)-2-(6-(pyrrolidin-1-yl)-3, 4-dihydroisoquinolin-1 (2*H*)-ylidene) ethanone (**11a**) (0.57 g); ((*Z*)-allyl (4-(2-(6-(pyrrolidin-1-yl)-3, 4-dihydroisoquinolin-1 (2*H*)-ylidene) acetyl) phenyl) carbonate (**11d**) (0.42 g)}, and dissolved in 5 mL dichloromethane. The solution was cooled to 0 °C, 0.91 mL diisopropylethylamine (0.65 g, 5.0 mmol, 5.0 equiv) was added.0.64 mL trifluorborane diethyl etherate (0.71 g, 5.0 mmol, 5.0 equiv) was added dropwise. The reaction mixture was stirred for 30 min at room temperature. The reaction mixture was concentrated and purified by flash column chromatography on silica gel using hexane/dichloromethane.

*(Z)-1-(4-((tert-butyldiphenylsilyl) oxy) phenyl)-2-(2-(difluoroboranyl)-6-(pyrrolidin-1-yl)-3, 4-dihydroisoquinolin-1 (2H)-ylidene) ethan-1-one (**12a**).*

Yield: 0.33 g (53%), yellow crystals, m.*p*. 261–262 °C (hexane-dichloromethane); ^1^ H NMR (500 MHz, CDCl_3_) *δ* 7.74 (t, *J* = 5.9 Hz, 2H, ArH), 7.73–7.69 (m, 4H, ArH), 7.58 (d, *J* = 8.8 Hz, 1H, ArH), 7.44 (dd, *J* = 8.4, 6.3 Hz, 2H, ArH), 7.37 (t, *J* = 7.3 Hz, 4H, ArH), 6.79 (d, *J* = 8.8 Hz, 2H, ArH), 6.45 (dd, *J* = 8.8, 2.3 Hz, 1H, HC=), 6.33 (s, 2H, ArH), 3.71 (t, *J* = 6.9 Hz, 2H, CH_2_), 3.37 (t, *J* = 6.5 Hz, 4H, CH_2_), 2.93 − 2.82 (m, 2H, CH_2_), 2.04 (dd, *J* = 7.9, 5.2 Hz, 4H, CH_2_), 1.12 (s, 9H, CH_3_) ppm; ^13^ C NMR (125 MHz, CDCl_3_) *δ* 189.2 (C = O), 174.0 (C=), 170.1 (C=), 167.5 (C=), 161.6 (C=), 158.6 (C=), 151.0 (C=), 139.8 (C=), 135.4 (C=), 132.4 (=CH), 130.1 (=CH), 129.1 (=CH), 128.5 (=CH), 127.9 (=CH), 119.7 (=CH), 114.2 (C=), 110.3 (=CH), 110.1 (=CH), 88.5 (=CH), 47.6 (CH_2_), 40,4 (CH_2_), 27.9 (CH_2_), 26.5 (CH_3_), 25.4 (CH_2_), 19.5 (CH_3_) ppm; ^19^ F NMR (282 MHz, CDCl_3_) *δ* −140.63–−140.75 (m, 1F, BF), −140.75–−140.87 (m, 1F, BF).; [M − F]^+^_measured_ = 601.2875, calcd. for C_37_H_39_BN_2_O_2_F: 601.2852.

*(Z)-allyl (4-(2-(2-(difluoroboranyl)-6-(pyrrolidin-1-yl)-3, 4-dihydroisoquinolin-1 (2H)-ylidene) acetyl) phenyl) carbonate (**12d**).*

Yield: 0.34 g (72%), yellow crystals, m.*p*. 180 °C (hexane-dichloromethane); ^1^H NMR (300 MHz, CDCl_3_) *δ* 7.99 (d, *J* = 7.5 Hz, 2H, NH), 7.64 (d, *J* = 8.7 Hz, 1H, ArH), 7.27 (d, *J* = 7.1 Hz, 2H, ArH), 6.48 (d, *J* = 11.8 Hz, 2H, ArH), 6.35 (s, 1H, HC=), 6.01 (dq, *J* = 10.2, 5.9 Hz, 1H, HC=), 5.44 (d, *J* = 17.1 Hz, 1H, HC=), 5.34 (d, *J* = 10.4 Hz, 1H, HC=), 4.75 (d, *J* = 5.7 Hz, 2H, CH_2_), 3.75 (t, *J* = 6.4 Hz, 2H, CH_2_), 3.44 − 3.34 (m, 4H, CH_2_), 2.91 (t, *J* = 6.6 Hz, 2H, CH_2_), 2.11 − 2.01 (m, 4H, CH_2_); ^13^C NMR (75 MHz, CDCl_3_) *δ* 186.0 (C = O), 160.4 (C = O), 153.5 (C=), 152.6 (C=), 150.1 (CH=), 139.9 (CH=), 138.7 (C=), 131.4 (C=), 128.4 (CH=), 127.7 (CH=), 120.8 (CH=), 119.8 (C=), 116.0 (C=), 110.6 (CH=), 110.5 (CH=), 85.5 (CH_2_), 69.4 (CH_2_), 47.8 (CH_2_), 39.1 (CH_2_), 29.2 (CH_2_), 25.7 (CH_2_) ppm; ^19^F NMR (282 MHz, CDCl_3_) *δ* −140.67 − −140.92 (m, 1F, BF), −140.93 − −141.19 (m, 1F, BF)., [M − F]^+^_measured_ = 447.1909, calcd. for C_25_H_25_BN_2_O_4_F: 447.1885.

*(Z)-2-(2-(difluoroboranyl)-6-(pyrrolidin-1-yl)-3,4-dihydroisoquinolin-1 (2H)-ylidene)-1-(4-hydroxyphenyl) ethan-1-one (**12c**).*

In a round bottom flask, 0.06 g (*Z*)-1-(4-((tert-butyldiphenylsilyl) oxy) phenyl)-2-(2-(difluoroboranyl)-6-(pyrrolidin-1-yl)-3, 4-dihydroisoquinolin-1 (2*H*)-ylidene) ethan-1-one (**15a**) (0.1 mmol) was dissolved in HPLC grade acetonitrile. Then, 0.32 g tetra-*n*-butylammonium fluoride (1 mmol, 10 equiv.) was added, and the reaction mixture was stirred at room temperature until full conversion checked by TLC and HPLC-MS. The reaction mixture was concentrated and purified by preparative TLC plate with dichloromethane (R_f_ = 0.37).

Yield: 0.010 g (26%), yellow crystals, m.*p*. 271–272 °C (dichloromethane); ^1^ H NMR (300 MHz, DMSO-*d_6_*) *δ* 10.19 (s, 1H, OH), 8.01 (d, *J* = 8.7 Hz, 1H, ArH), 7.93 (d, *J* = 8.3 Hz, 2H, ArH), 6.87 (d, *J* = 8.3 Hz, 2H, ArH), 6.75 (s, 1H, ArH), 6.56 (d, *J* = 8.5 Hz, 1H, ArH), 6.51 (s, 1H, HC=), 3.60 − 3.50 (m, 2H, CH_2_), 3.42 − 3.33 (m, 4H, CH_2_, overlap with water), 2.91 − 2.82 (s, 2H, CH_2_), 2.04 − 1.93 (m, 4H, CH_2_) ppm; ^13^ C NMR (125 MHz, CDCl_3_) *δ* 166.8 (C = O), 161.6 (C=), 161.2 (C=), 151.4 (C=), 140.0 (C=), 130.6 (C=), 129.3 (=CH), 125.1 (C=), 115.9 (=CH), 113.7 (C=), 110.9 (=CH), 110.7 (=CH), 88.4 (=CH), 47.9 (CH_2_), 40.3 (CH_2_, overlap with DMSO-*d_6_*), 27.6 (CH_2_), 25.4 (CH_2_) ppm; ^19^ F NMR (282 MHz, CDCl_3_) δ −137.18 (d, *J* = 16.0 Hz, 1F, BF), −137.32 (d, *J* = 13.3 Hz, 1F, BF) ppm; [M − F]^+^_measured_ = 363.1683, calcd. for C_21_H_21_BN_2_O_2_F: 363.1674.

## 3. Results and Discussion

**Scheme 1:**
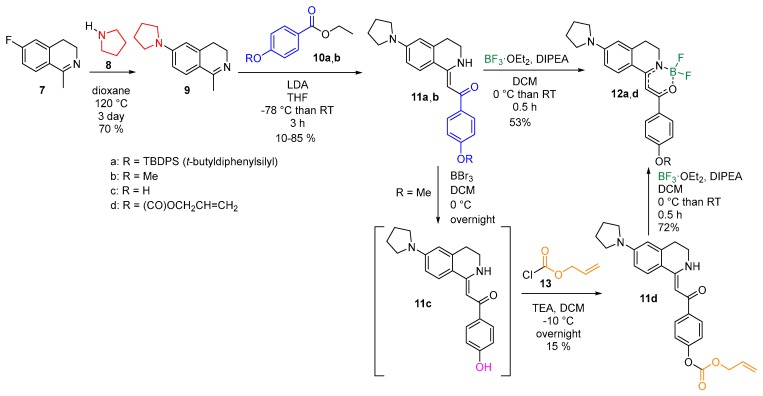
The synthesis of the boroisoquinoline chemodosimeters.

The common intermediate for the two designed chemodosimeters was planned to be 6-fluoro-1-methyl-3, 4-dihydroisoquinoline (**9**) that has been synthesized by following literature procedures [20]. 3-Fluorophenyl ethaneamine was acetylated, and a modified Bishler–Napieralski reaction resulted in compound **7** with good yields. The fluorinated isoquinoline (**7**) was then transformed to 6-pyrrolidinyl-1-methyl-3, 4-dihydroisoquinoline (**9**) by an aromatic nucleophilic substitution with pyrrolidine followed by an LDA (lithium diisopropylamide)-promoted acylation of the methyl group resulting in 1-(4-((*tert*-butyldiphenylsilyl) oxy) phenyl)-2-(6-(pyrrolidin-1-yl)-3, 4-dihydroisoquinolin-1 (2*H*)-ylidene) ethan-1-one (**11a**) and 1-(4-methoxyphenyl)-2-(6-(pyrrolidin-1-yl)-3, 4-dihydroisoquinolin-1 (2*H*)-ylidene) ethan-1-one (**11b**) with various yields. For this reaction step, an imine-enamine tautomerization is proposed involving the methyl group (see Supplementary Material). For the fluoride chemodosimeter (**12a**), the addition of the silylated benzoic acid ester (**10a**) was a straightforward approach, while, in the case of the Pd (0) sensing compound (**12d**), the allyloxycarbonyl group was not compatible with the strong basic conditions. Thus, at first, the methoxy group of **11b** was demethylated with boron tribromide resulting in a phenolic compound **11c** that was equipped **by** the allyloxycarbonyl recognition element in an acylation reaction with low yield. The reaction of **11a**,**d** with boron trifluoride diethyl etherate in the presence of DIPEA (diisopropyl ethaneamine) was the final step resulting in the target compounds 1-(4-((*tert*-butyldiphenylsilyl) oxy) phenyl)-2-(2-(difluoroboranyl)-6-(pyrrolidin-1-yl)-3, 4-dihydroisoquinolin-1(2*H*)-ylidene) ethan-1-one (**12a**) and (*Z*)-allyl (4-(2-(2-(difluoroboranyl)-6-(pyrrolidin-1-yl)-3, 4-dihydroisoquinolin-1 (2*H*)-ylidene) acetyl) phenyl) carbonate (**12d**) (Scheme 1) with mediocre yields.

After the successful synthesis of the two chemodosimeters (**12a**,**d**), we have investigated thoroughly their photophysical properties and their sensitivity and selectivity against the corresponding analytes. The photophysical properties were measured in acetonitrile (Table 1, Supplementary Figure S1). The molar absorbance coefficient corresponds to the absorbance maximum wavelength. One might conclude that **12a**,**d** has emission in the green range of the spectrum with a high Stokes-shift. Excellent quantum yield and acceptable ε have been measured for **12a.** However, although the quantum yield for **12d** is low, with the high ε, it can still be considered as having appropriate brightness.

### 3.1. The Fluoride Chemodosimeter

**Scheme 2:**
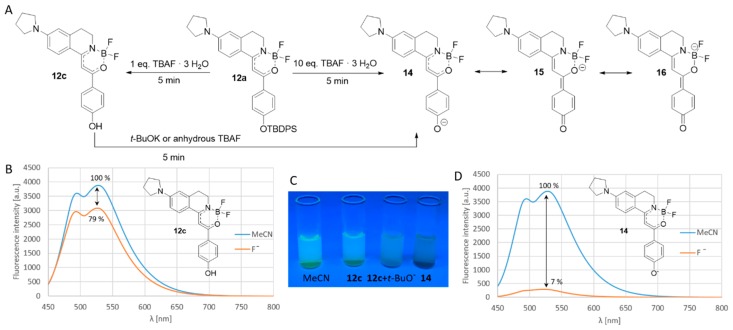
(**A**) proposed mechanism of fluoride detection by **12a**; (**B**) emission spectra of **12c**; (**C**) visible changes in the reactions of **12a**; (**D**) emission spectra of **14**.

Firstly, we have treated the solution of **12a** in acetonitrile with one equivalent of crystalline tetrabutylammonium fluoride (TBAF∙3 H_2_O), and observed a decrease in the fluorescence intensity. Applying a larger amount of 10 equivalents fluoride completely turned off the fluorescence. Based on the basicity of the fluoride anion in organic solvents, it is proposed that, after the desilylation, a phenolate would be formed, but, in the presence of water, it is protonated leading to weak fluorescence. Applying a large excess of fluoride, the phenolate stays deprotonated. In this case, the negatively charged phenolate and its quinoidal mesomeric structure disrupt the conjugation of the phenyl group with the boranyl moiety leading to the loss of the emission. In order to prove this theory, we have isolated the desilylated phenolic compound (**12c**) and we have proven its structure by HPLC-MS and NMR techniques. **12c** showed weak fluorescence, and, after the deprotonation with potassium *tert*-butylate, the emission of the substance was switched off. Notably, in the presence of dry TBAF in the absence of water, the emission was completely turned off as well (Scheme 2).

**Figure 4:**
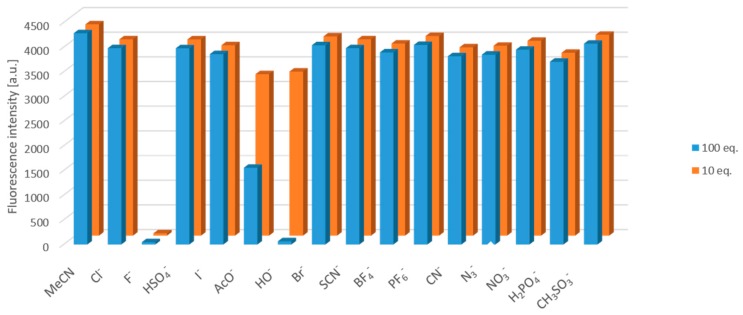
Fluorescence intensity changes caused by the anions at the wavelength of the maximal intensity after 5 min.

**Figure 5:**
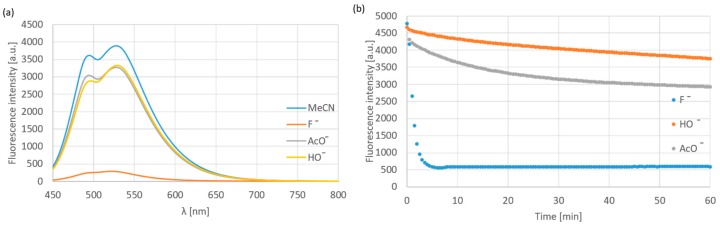
(**a**) fluorescence spectra obtained in the presence of 10 equivalents fluoride, acetate and hydroxy; (**b**) changes of the emission intensity during 60 min at 528 nm in the presence of the same anions.

The anion-selectivity of compound **12a** was tested with 100 equiv. tetrabutylammonium salts in acetonitrile. The applied anions were halogens (Cl^−^, Br^−^, I^−^), HSO_4_^−^, OAc^−^, OH^−^, SCN^−^, BF_4_^−^, PF_6_^−^, CN^−^, N_3_^−^, H_2_PO_4_^−^ and MeSO_3_^−^. It was found that, besides the fluoride, the fluorescence intensity was turned off in the presence of OH^−^ and decreased by OAc^−^ (Figure 4). However, applying 10 equiv. excess of the anions, the selectivity was proven to be complete (Figure 4 and Figure 5a); only the fluoride turned off the emission rapidly, while the effect of OH^−^ and AcO^−^ was not significant even after a long time (Figure 5b).

**Figure 6:**
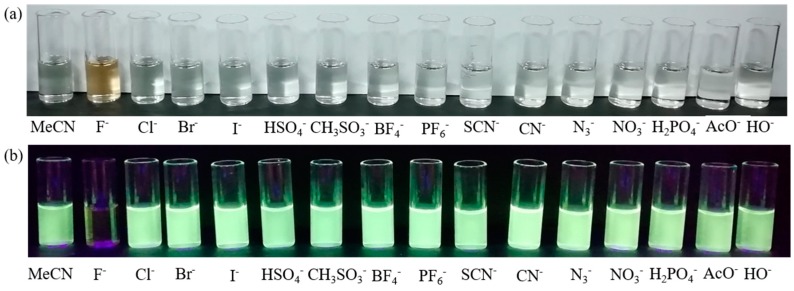
(**a**) photo of the chemodosimeter in acetonitrile with the anions without excitation; (**b**) photo of the chemodosimeter in acetonitrile with the anions applying excitation at 366 nm.

The presence of fluoride shows an effect not only on the emission, but on the absorption as well. The effect is easily visible in the absorbance spectrum (Supplementary Figure S2) and with the naked eyes as well, which might be advantageous by field measurements and tests (Figure 6).

**Figure 7:**
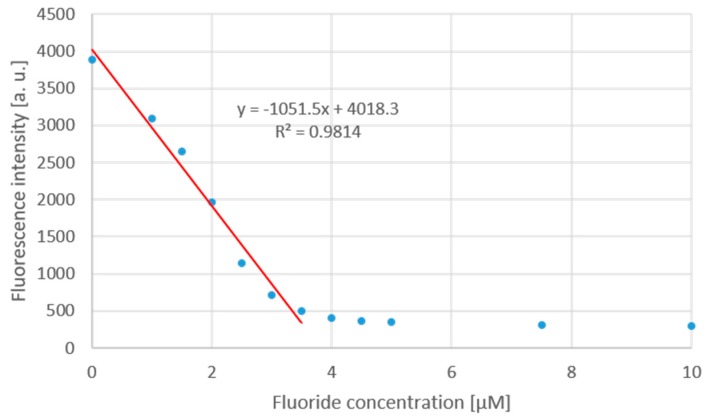
The loss of fluorescence intensity as a function of the fluoride concentration.

A titration curve has been recorded with TBAF∙3 H_2_O and the solution of chemodosimeter (1 µM, **12a**) (Figure 7). The fluorescence intensity decreased linearly until TBAF concentration of 3.5 µM, and thereafter the fluorescence turned off completely.

**Figure 8:**
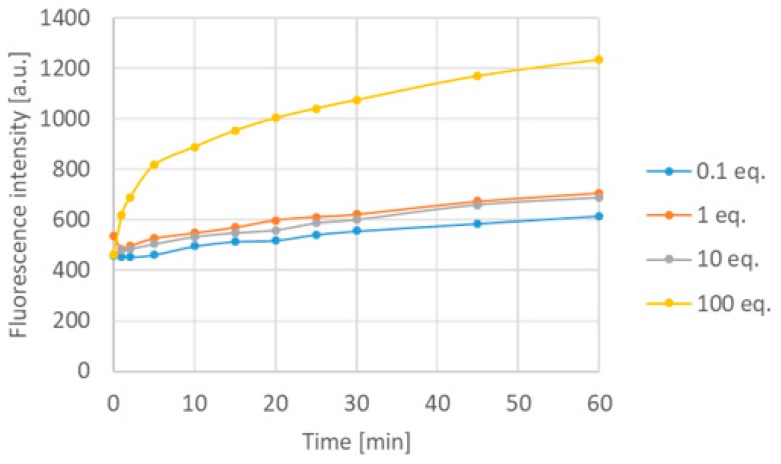
Time-dependency of the emission of **12a** in aqueous solution at 489 nm.

Considering that the chemodosimeters are used for prompt measurements on the field for examining the quality of water (like open water sources, springs, wells, and waste water), it is necessary to check the efficacy of the **12a** chemodosimeter in water solution. However, the boroisoquinolines underwent aggregation in pure water; therefore, PBS buffer (50 mM phosphate, pH = 7.4) with 0.1% SDS was applied. It should be mentioned that investigating absorbance and fluorescence spectra of **12a** in dimethyl formamide, dimethyl acetamide and DMSO, the absorbance and fluorescence intensity decreased to ~40% (see Supplementary Figure S1), which can be addressed to the polarity of the media. The concentration of the chemodosimeter was set to 10 µM, and the change of the emission spectra was examined by different fluoride concentrations. Firstly, it was observed that the medium is significantly influencing the function of the chemodosimeter; in particular, in aqueous solution, the emission intensity of the fluorescent probe increased. Notably, this is more favorable than the switch-off mechanism. However, the emission maximum moved to the direction of blue light. It is supposed that, in water, the fluoride ion is a less strong base; furthermore, the phenolic OH-group can be protonated in the aqueous medium, eliminating the negative charge responsible for turning off the fluorescence. Although the addition of 0.1, 1 and 10 equivalents of the analyte did not cause significant emission growth over a 60-minute period, but 100 equivalents of the fluoride doubled the emission intensity at the maximum wavelength after 5 min (Figure 8).

### 3.2. The Pd (0) Chemodosimeter

**Figure 9:**
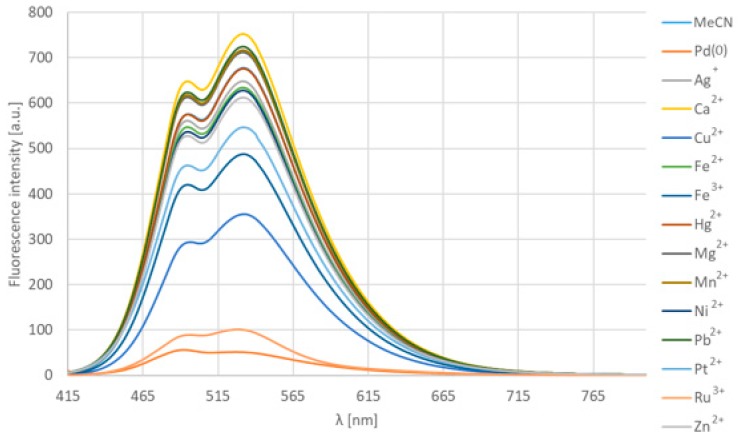
The emission spectra of **12d** in the presence of 100 equivalents of metals and different cations in acetonitrile at room temperature after 5 min.

The same methodology was followed in this case as that for the fluoride chemodosimeter. We have treated compound **12d** with 100 equiv. of metals or metal cations besides Pd_2_dba_3_ [tris(dibenzylideneacetone)dipalladium], in particular Ag^+^, Ca^2+^, Cu^2+^, Fe^2+^, Fe^3+^, Hg^2+^, Mg^2+^, Mn^2+^, Ni^2+^, Pb^2+^, Pt^2+^, Ru^3+^, Zn^2+^ (Figure 9). In most cases, chloride salts were available, except of Hg and Pb, which were used as an iodide and nitrate salt, respectively. In general, there were no spectral changes to observe, only in the case of Pd (0) that decreased the intensity and shifted the emission maximum to a lower range (from 532 nm to 491 nm). However, by several other metal ions (Ru^3+^, Cu^2+^, Pt^2+^, Fe^3+^), the decrease of the intensity was observed as well, but there was no shift. This effect was caused by the absorption of the exciting light by the transition metals [21,22]. Based on HPLC-MS measurements, it has been proven that no chemical reaction occurred in the presence of the latter cations. On the contrary, Pd (0) caused the leave of the allyloxy group with full conversion, resulting in compound **12c** together with turning off the emission.

**Scheme 3:**
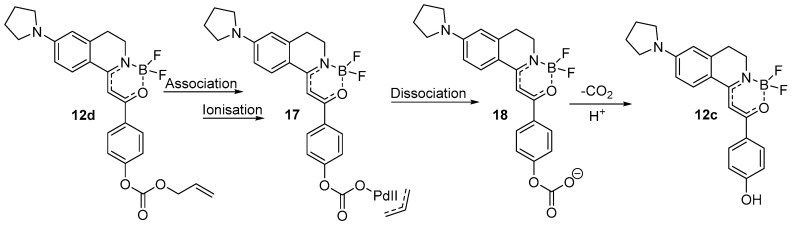
The supposed mechanism for the Pd (0) detection of **12d**.

The mechanism of action of Pd (0) with allyloxy recognition functional groups was proposed in numerous earlier cases similarly [17], thus it might be accepted that firstly the Pd (0) metal associates with the electron system of the allyloxy group cleaving the O-C bond resulting in a propylene released. Meanwhile, Pd (0) turns to its oxidized form Pd (II). After the dissociation of the metal, a CO_2_ molecule is able to leave intermediate **18** by decarboxylation resulting in compound **12c** (Scheme 3).

**Figure 10:**
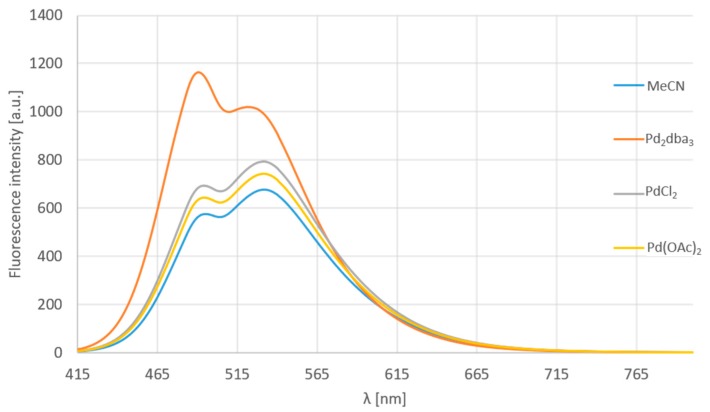
The emission spectra of **12d** in the presence of 10 equivalent different palladium compounds.

Further examination of the selectivity with different palladium salts and Pd (0) dibenzylideneacetone-complex was accomplished (Figure 10). Moreover, Ni (0) and Pt (0) was added to these confirmative experiments. One might observe that substance **12d** is not sensible for Pd (II) salts (PdCl_2_, Pd(OAc)_2_), but selectively, only for the Pd (0) complex. The addition of Ni (0) and Pt (0) caused a significant drop in the fluorescence, but no change in the shape of the spectrum. Notably, MS measurements have proven that Pd_2_dba_3_ was the only agent causing the chemical reaction responsible for the considerable change in the emission spectrum. Ten equivalents of the metals and salts were used with an incubation time of 5 min. The observed intensity loss is associated with the light absorption of the metal.

**Figure 11:**
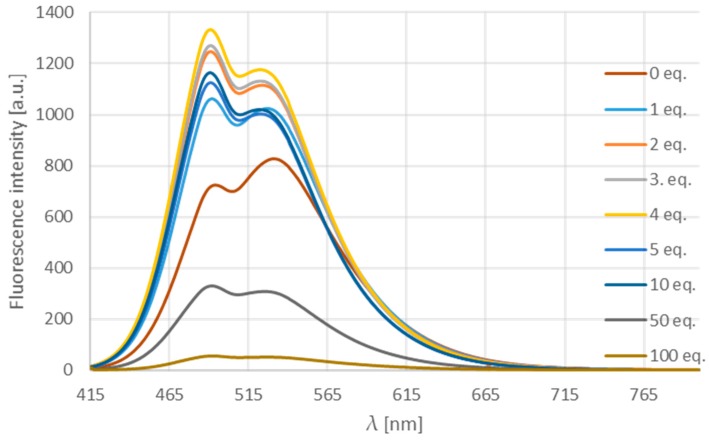
The emission spectra of **12d** in the presence of different equivalents Pd_2_dba_3._

The titration of the acetonitrile solution of compound **12d** with Pd_2_dba_3_ (Figure 11) showed that, until four equivalents the intensity grows, while, applying a larger excess, it decreases possibly due to the absorption of the light by the metal in high concentration.

**Figure 12:**
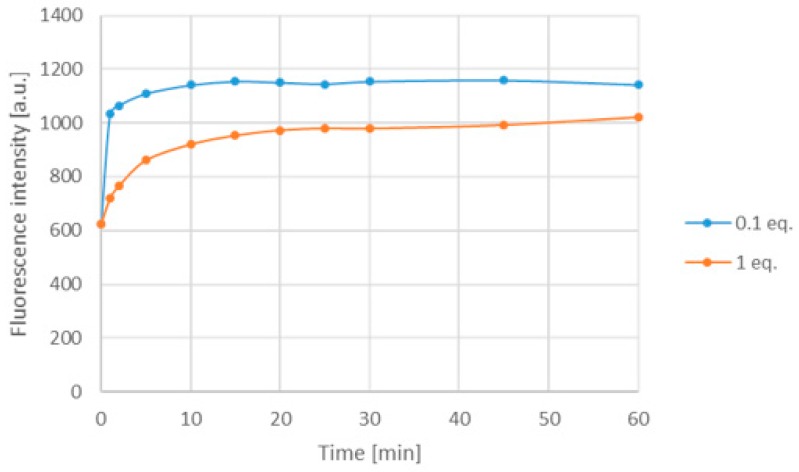
The change of the emission of **12d** in water at 537 nm.

Investigating absorbance and fluorescence spectra of **12d** in polar solvents, in particular, dimethyl formamide, dimethyl acetamide and DMSO, the absorbance and fluorescence intensity did not show any change (see Supplementary Figure S3). The emission spectrum was measured in the presence of Pd_2_dba_3_ in aqueous solution as well. In this case, the metal light absorption was similarly significant at 10 equivalents excess; therefore, the amount of Pd_2_dba_3_ was decreased to 10% or 1 equivalent resulting in an increase in the fluorescence after 5 min and forming a saturation curve in both cases (Figure 12).

## 4. Conclusions

Based on the development of a family of new fluorophores, we have successfully applied appropriately substituted derivatives on the field of analytical detection. New fluoride- and Pd (0)-sensing molecules were synthesized with good overall yields in multistep syntheses. Both chemodosimeters showed excellent selectivity toward the corresponding analyte. The fluoride-selective boroisoquinoline showed some restriction on the concentration, while the other one was able to detect Pd (0) selectively towards Pd with other oxidation state even at catalytic concentration. Moreover, the boroisoquinoline chemodosimeters were successfully applied in aqueous media as well extending the possible application fields.

## Figures and Tables

**Table 1:** Photophysical properties of the **12a,d** measured in acetonitrile.

Entry	Compound	$\lambda_{abs}^{max}\;(\mathrm{nm})$	$\lambda_{em}^{max}\;(\mathrm{nm})$	Δλ (nm)	ε (M^−1^ cm^−1^)	$\Phi_{F}$
1	**12a**	398	527	129	18,671	0.88
2	**12d**	408	532	124	82,101	0.10

## Supplementary Materials

The following are available online at https://www.mdpi.com/1996-1944/13/1/199/s1, synthesis methods for compounds known in the literature, absorbance, excitation and emission spectra, ^1^ H and ^13^ C spectra.
