# Supplementary material for: Application of Boroisoquinoline Fluorophores as Chemodosimeters for Fluoride Ion and Pd (0)
Source: Materials (Basel). 2020 Jan 2;13(1):199. doi: 10.3390/ma13010199 (PMC6981940; doi:10.3390/ma13010199)
Supplement: Supplementary file 1 [file materials-13-00199-s001.pdf]

materials

Supplementary Materials

Application of Boroisoquinoline Fluorophores as Chemodosimeters for Fluoride Ion and Pd (0)

Dénes Sóvári, György Miklós Keserű and Péter Ábrányi-Balogh *

Research Centre for Natural Sciences, Institute of Organic Chemistry, Medicinal Chemistry Research Group, POB 286, 1519 Budapest, Hungary; sovari.denes@ttk.mta.hu (D.S.); keseru.gyorgy@ttk.mta.hu (G.M.K.)
* Correspondence: abranyi-balogh.peter@ttk.mta.hu; Tel.: +36-1-382-6961

1. Synthesis Methods for Compounds Known in the Literature

N-[2-(3-Fluorophenyl)ethyl]acetamide [1]

In a three-necked round bottom flask 3.9 g 2-(3-fluorophenyl)ethan-1-amine (28 mmol) and 5.1 mL triethyl-amine (3.8 g, 36.4 mmol, 1.3 equiv) was dissolved in 50 mL dichloromethane. To this solution was added dropwise 2.4 mL acetyl chloride (2.6 g, 34.0 mmol, 1.2 equiv) and the mixture was stirred at room temperature for 30 minutes. The reaction mixture was quenched with 50 mL water. The layers were separated, and the organic layer was washed with 40 mL 1 M HCl and then 40 mL 5% Na_2CO_3 solution, dried over $MgSO_4$, filtered and evaporated under reduced pressure.

Yield: 4,2 g (83%), yellow oil; ^{1}H NMR (500 MHz, $CDCl_3$) δ 7.25 (dd, *J* = 13.9, 7.9 Hz, 1H, ArH), 6.96 (d, *J* = 7.6 Hz, 1H, ArH), 6.90 (d, *J* = 6.5 Hz, 1H, ArH), 5.63 (s, 1H, NH), 3.49 (dd, *J* = 13.1, 6.8 Hz, 2H, CH_2), 2.80 (t, J = 7.0 Hz, 2H, CH_2), 1.93 (s, 3H, CH_3) ppm.

6.-F.luoro-1-methyl-3,4-dihydroisoquinoline (7) [2]

In a three-necked round bottom flask 3.4 g *N*-[2-(3-fluorophenyl)ethyl]acetamide(18.9 mmol) was dissolved in 100 mL dichloromethane and 1.8 mL oxalyl chloride (2.6 g, 20.7 mmol, 1.1 equiv) was added dropwise under nitrogen atmosphere. The solution was stirred at room temperature for 30 minutes. Then the reaction mixture was cooled below –10 °C, and 3.7 g $FeCl_3$ (22.7 mmol, 1.2 equiv) was added. The mixture was stirred at room temperature overnight. Aqueous 2M HCl (10 mL, 20.0 mmol) was added to quench the reaction. The mixture was stirred at room temperature for 1 h, then the layers were separated. The organic layer was washed with brine, and dried over $MgSO_4$), filtered, and the solvent was removed in vacuo. The oxalyl adduct was dissolved in 180 mL methanol, 9.5 mL concentrated sulphuric acid was added, and stirred at reflux overnight. The next morning the reaction mixture was allowed to cool room temperature, and the solvent was removed *in vacuo*. The crude material was dissolved in ethyl acetate, and washed with water. The aqueous layer was basified with 10 % sodium hydroxide solution, and extracted with ethyl acetate. The combined organic phases were dried ($MgSO_4$), filtered and the solvent was removed in vacuo.

Yield: 2.3 g (76%), tawny oil; ^{1}H NMR (500 MHz, $CDCl_3$) δ 7.56 (dd, *J* = 8.5, 5.5 Hz, 1H, ArH), 7.01 (td, *J* = 8.5, 2.5 Hz, 1H, ArH), 6.93 (dd, *J* = 8.6, 2.2 Hz, 1H, ArH), 3.73 (dd, *J* = 12.8, 5.2 Hz, 2H, CH_2), 2.83–2.76 (m, 2H, CH_2), 2.49 (s, 3H, CH_3) ppm.

1.-m.ethyl-6-(pyrrolidin-1-yl)-3,4-dihydroisoquinoline (9) [2]

In a sealed tube 4.4 g 6-fluoro-1-methyl-3,4-dihydroisoquinoline (7) (27.0 mmol) and 10 mL pyrrolidine (8 (11.5 g, 162 mmol, 6 equiv) were dissolved in 10 mL dioxane. The reaction mixture was heated at 80 °C for 3 days. The residue concentrated under reduced pressure, and purified with steam distillation. After all of the excess pyrrolidine was distilled, the leftover was dissolved in aqueous HCl, then extracted with dichloromethane (5 × 50 mL). For NMR purpose the crude product purified with reverse phase column chromatography using acetonitrile/water.

Yield: 4.0 g (70 %), yellow crystals, m. p. 47–48 °C (water-MeCN); ^{1}H NMR (500 MHz, $CDCl_3$) δ 7.45 (d, *J* = 8.9 Hz, 1H, ArH), 6.41 (dd, *J* = 8.9, 2.3 Hz, 1H, ArH), 6.28 (s, 1H, ArH), 3.66 (t, *J* = 6.8 Hz, 2H, CH_2), 3.38–3.28 (m, 4H, CH_2), 2.84 (t, J = 7.6 Hz, 2H, CH_2), 2.59 (s, 3H, CH_3), 1.97 (dt, *J* = 17.0, 6.7 Hz, 4H, CH_2) ppm.

Ethyl 4-((tert-butyldiphenylsilyl)oxy)benzoate (10a)[3]

In a round bottom flask 1.0 g ethyl-4-hydroxybenzoate (5.9 mmol), 0.8 g imidazole (11.8 mmol, 2 equiv.) and 3.1 mL *tert*-butyl(chloro)diphenylsilane (3.2 g, 11.8 mmol, 2 equiv.) were dissolved in 20 mL dichloromethane. The reaction mixture was stirred at room temperature overnight. The next day it was evaporated, the leftover was dissolved in 50 mL ethyl acetate and extracted with 20 mL concd. Na_2CO_3 solution. The organic layer was dried ($MgSO_4$), filtered and the solvent was removed *in vacuo*. The residue was purified by flash chromatography on silica gel using hexane/ethyl acetate.

Yield: 1.8g (75%), colourless oil; ^{1}H NMR (500 MHz, $CDCl_3$) δ 7.81 (d, *J* = 8.7 Hz, 2H, ArH), 7.70 (dd, *J* = 8:0, 1.2 Hz, 4H, ArH), 7.43 (d, *J* = 7.6 Hz, 4H, ArH), 7.37 (t, *J* = 7.5 Hz, 2H, ArH), 6.78 (d, *J* = 8.7 Hz, 2H, ArH), 4.30 (d, *J* = 7.1 Hz, 2H, CH_2), 1.33 (t, *J* = 7.1 Hz, 3H, CH_3), 1.11 (s, 12H, CH_3) ppm.

Ethyl 4-methoxybenzoate (10b)[4]

In a sealed tube 2.0 g ethyl-4-hydroxybenzoate (12.0 mmol), 10 mL acetone, 1.8 g K_2CO_3 (13.2 g, 1.1 equiv.), 1.1 mL iodomethane (2.6 g, 18.0 mmol, 1.5 equiv.) were loaded. The reaction mixture was heated at 60 °C. After 4 h the reaction was completed verified by LCMS. Then it was filtered and concentrated *in vacuo*. The residue was purified by flash chromatography on silica gel using hexane/ethyl acetate.

Yield: 1.7 g (79%), colourless oil; ^{1}H NMR (500 MHz, $CDCl_3$) δ 8.01–7.97 (m, 2H, ArH), 6.92–6.88 (m, 2H, ArH), 4.34 (q, *J* = 7.1 Hz, 2H, CH_2), 3.84 (s, 3H, OCH_3), 1.37 (t, *J* = 7.1 Hz, 3H, CH_3) ppm.

2. Proposed Mechanism for the Synthesis of 12a and 12d

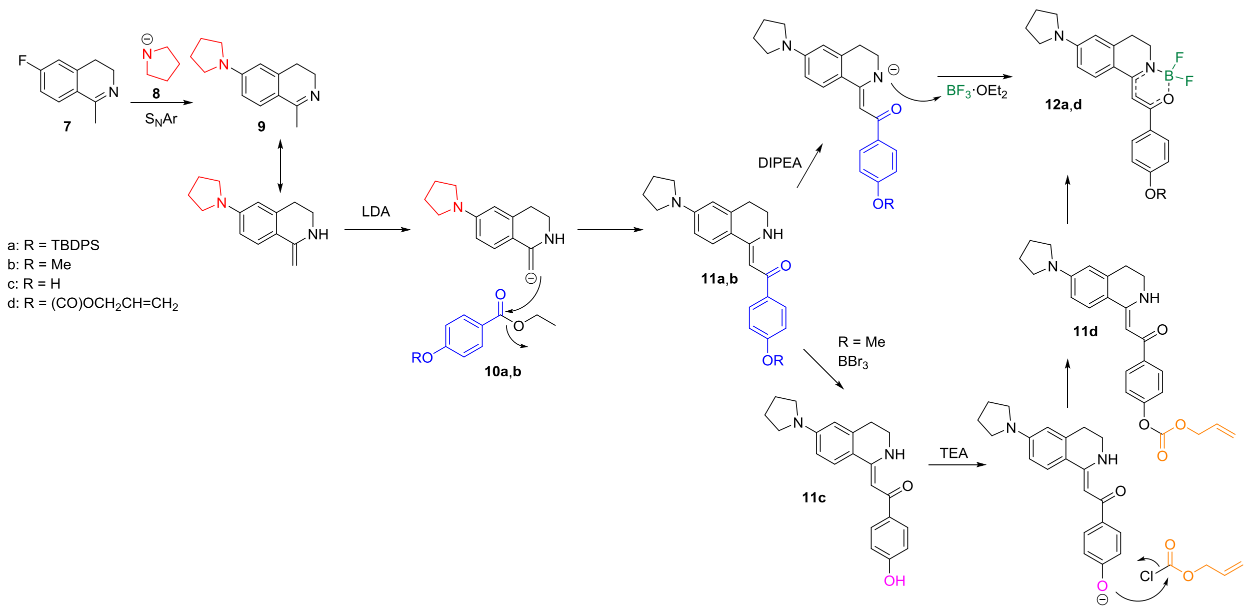

3. Absorbance, Excitation and Emission Spectra

Spectra of (Z)-1-(4-((tert-butyldiphenylsilyl)oxy)phenyl)-2-(2-(difluoroboranyl)-6-(pyrrolidin-1-yl)-3,4-dihydroisoquinolin-1(2H)-ylidene)ethan-1-one (12a)

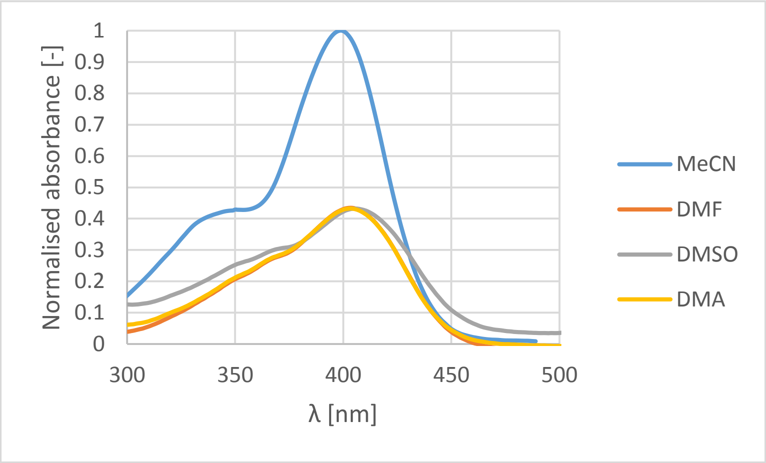

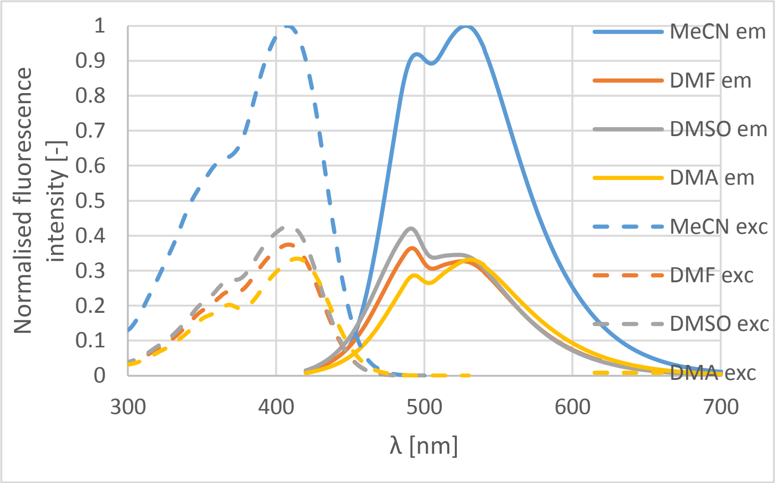

Figure S1. Absorbance, excitation and emission spectra of 12a in different solvents normalised to the highest values.

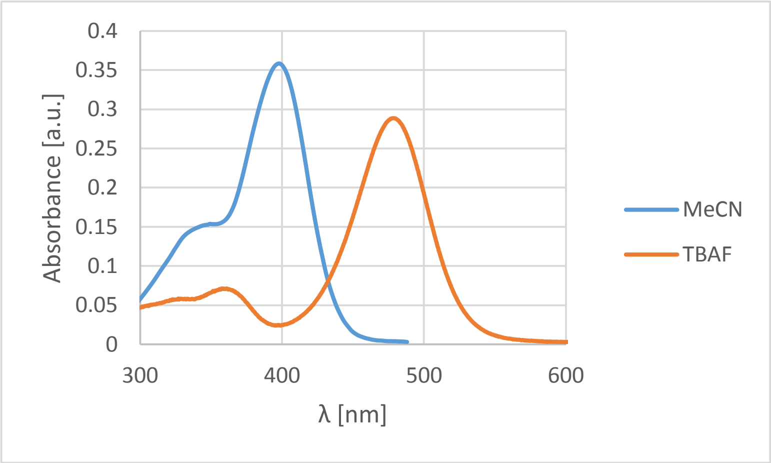

Figure S2. Change of absorbance spectra of 12a after addition of TBAF.

Spectra of (Z)-allyl (4-(2-(2-(difluoroboranyl)-6-(pyrrolidin-1-yl)-3,4-dihydroisoquinolin-1(2H)-ylidene)acetyl)phenyl) carbonate (12d)

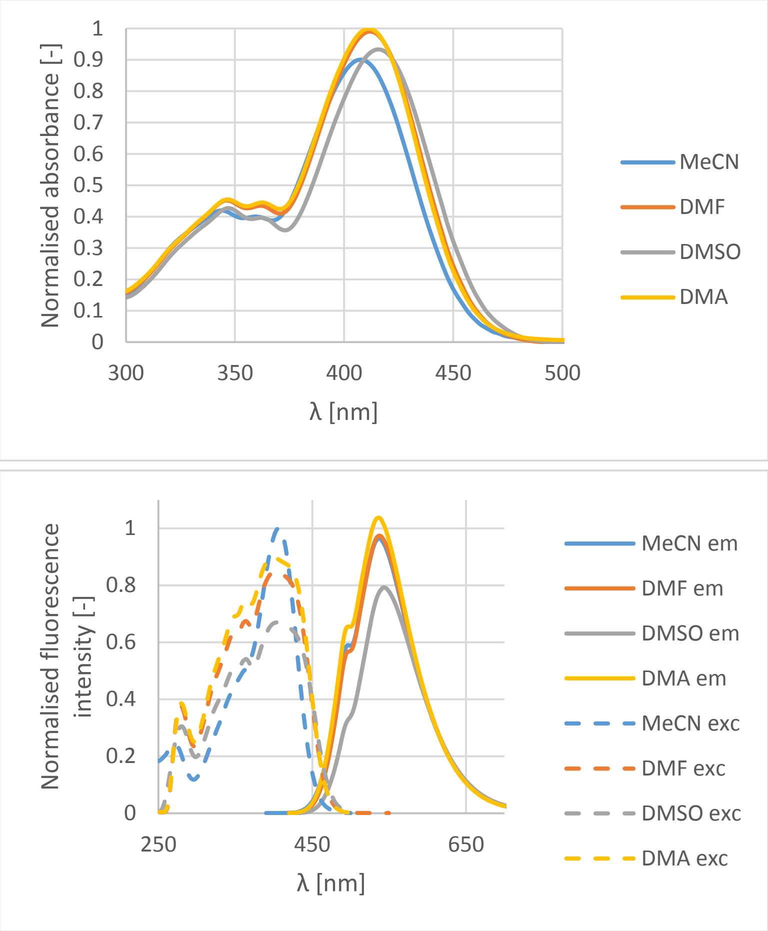

Figure S3. Absorbance, excitation and emission spectra of 12d in different solvents normalised to the highest values.

4. ^{1}H and ^{13}C Spectra

(Z)-1-(4-((tert-butyldiphenylsilyl)oxy)phenyl)-2-(6-(pyrrolidin-1-yl)-3,4-dihydroisoquinolin-1(2H)-ylidene)ethanone (11a)

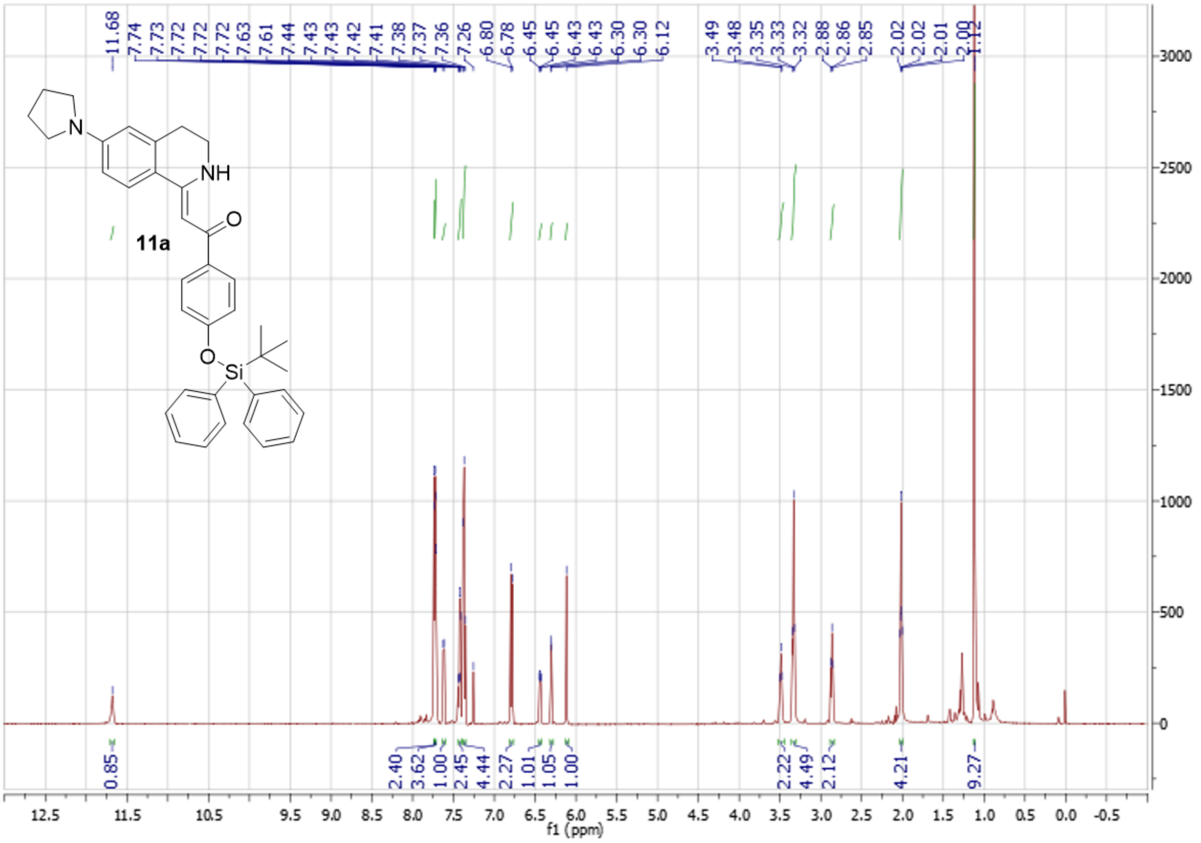

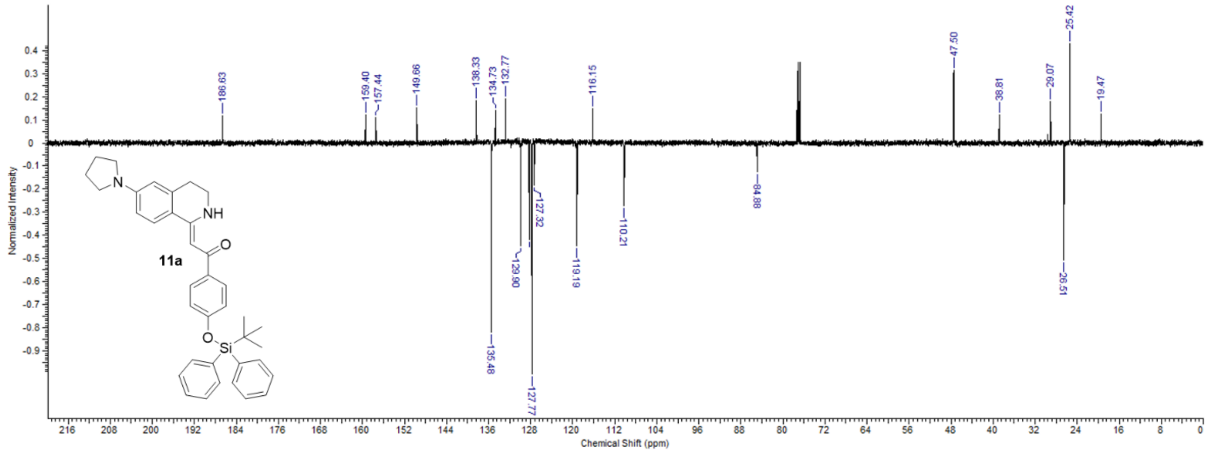

(Z)-1-(4-methoxyphenyl)-2-(6-(pyrrolidin-1-yl)-3,4-dihydroisoquinolin-1(2H)-ylidene)ethanone (11b)

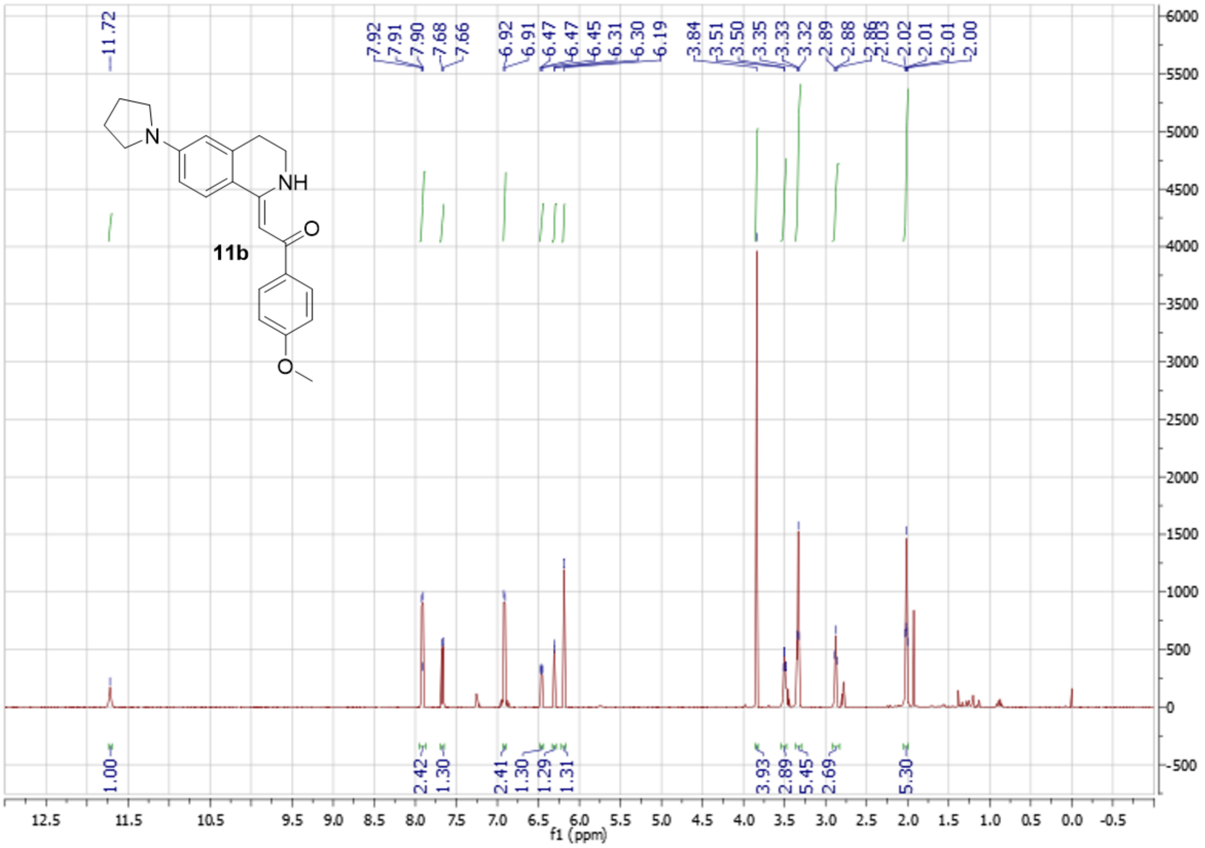

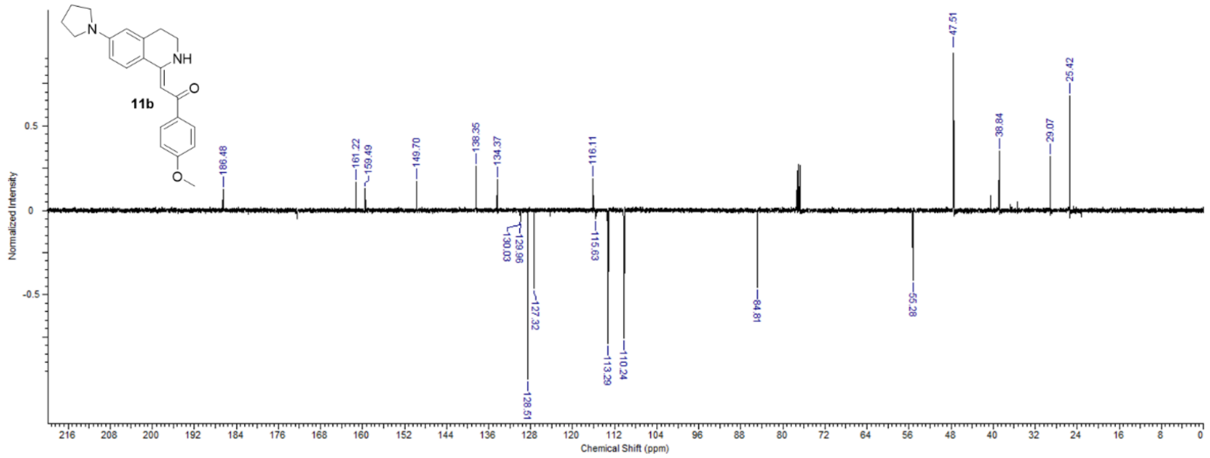

(Z)-allyl (4-(2-(6-(pyrrolidin-1-yl)-3,4-dihydroisoquinolin-1(2H)-ylidene)acetyl)phenyl) carbonate (11d)

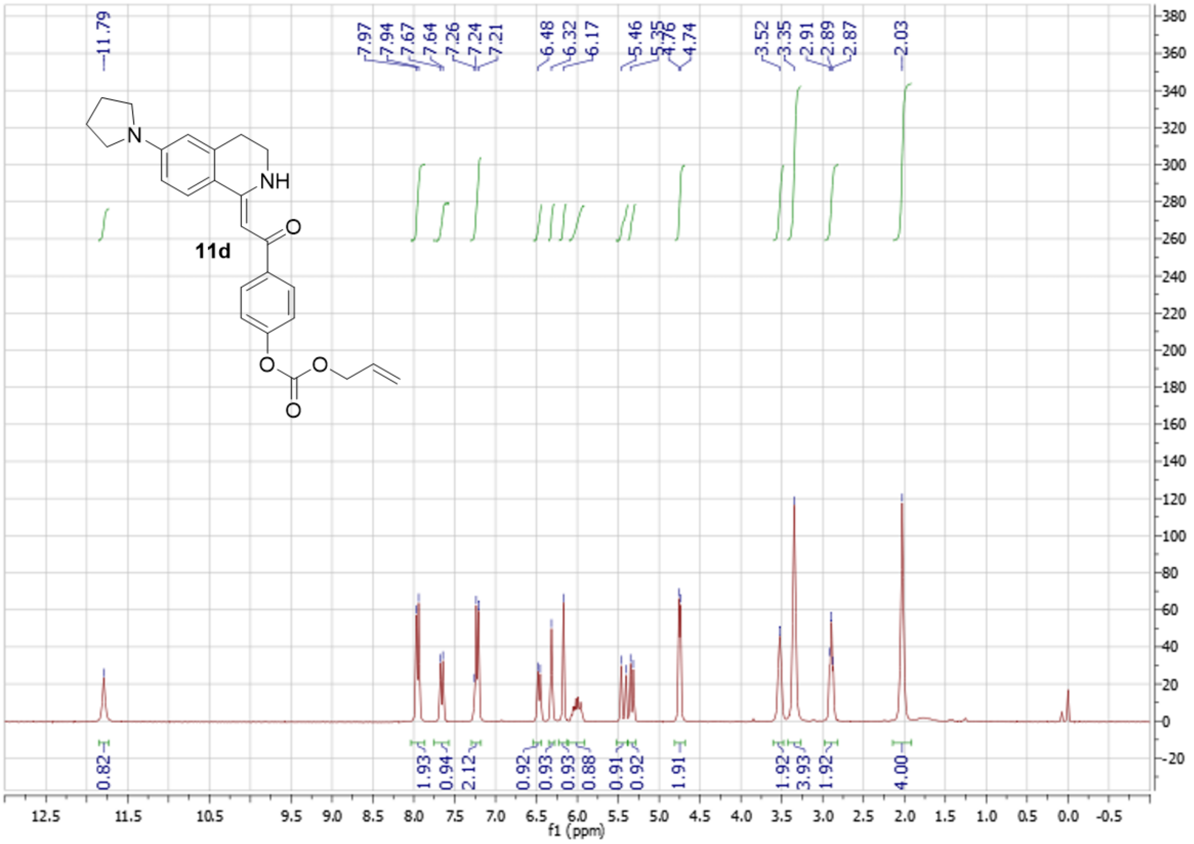

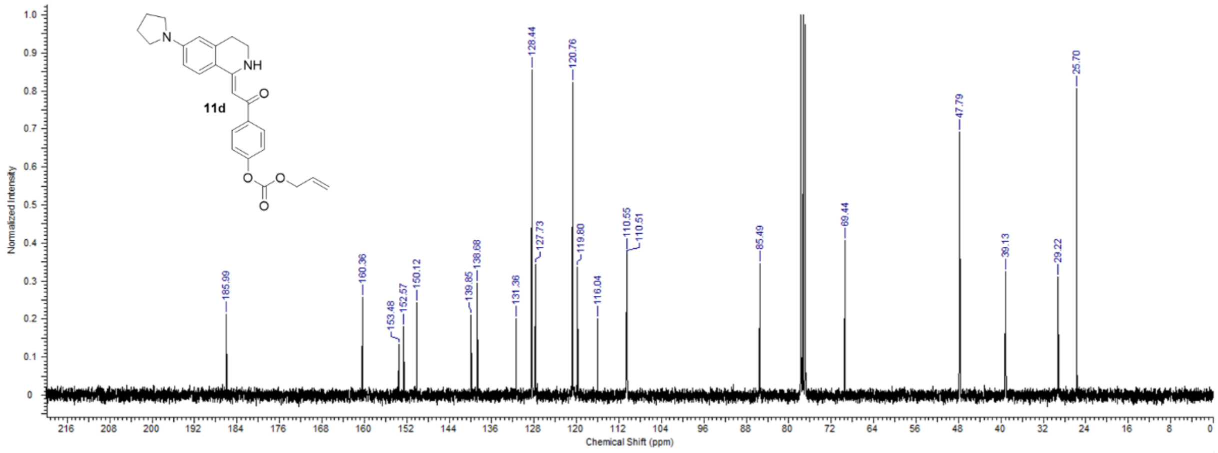

(Z)-1-(4-((tert-butyldiphenylsilyl)oxy)phenyl)-2-(2-(difluoroboranyl)-6-(pyrrolidin-1-yl)-3,4-dihydroisoquinolin-1(2H)-ylidene)ethan-1-one (12a)

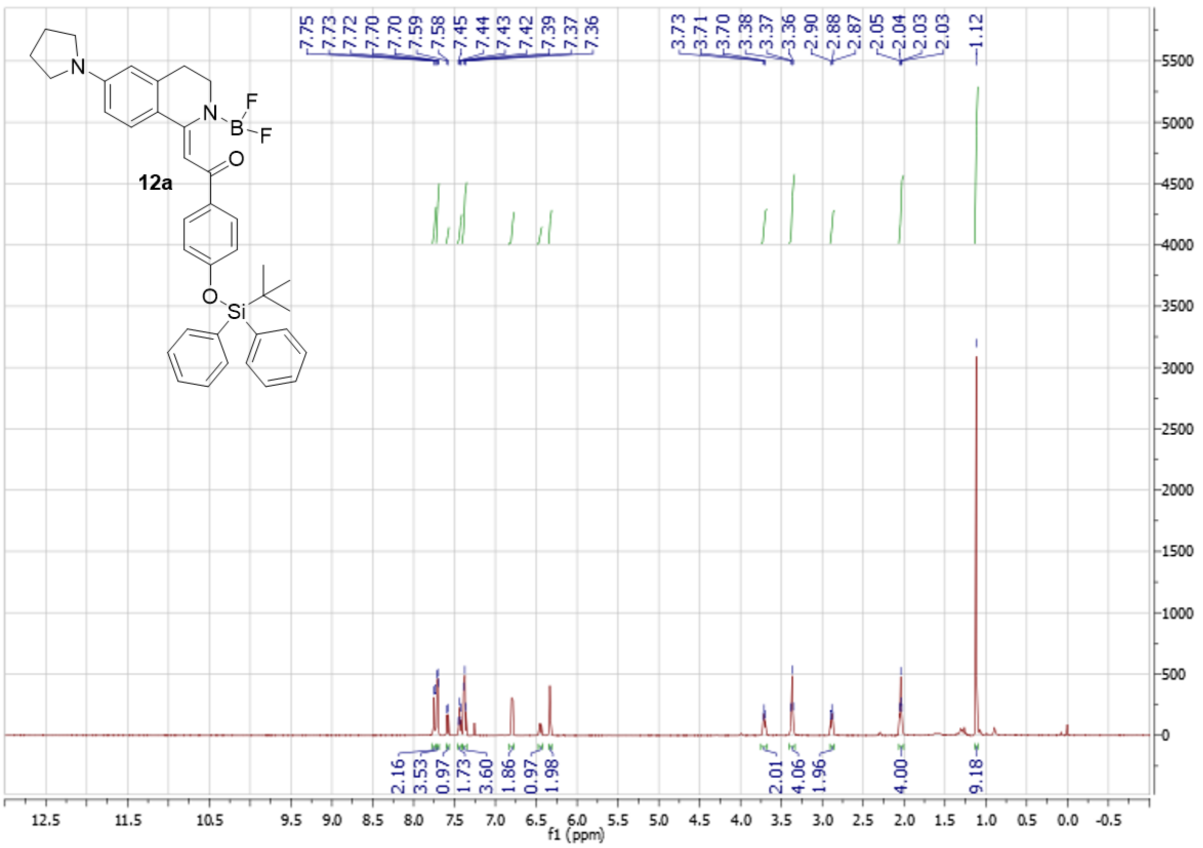

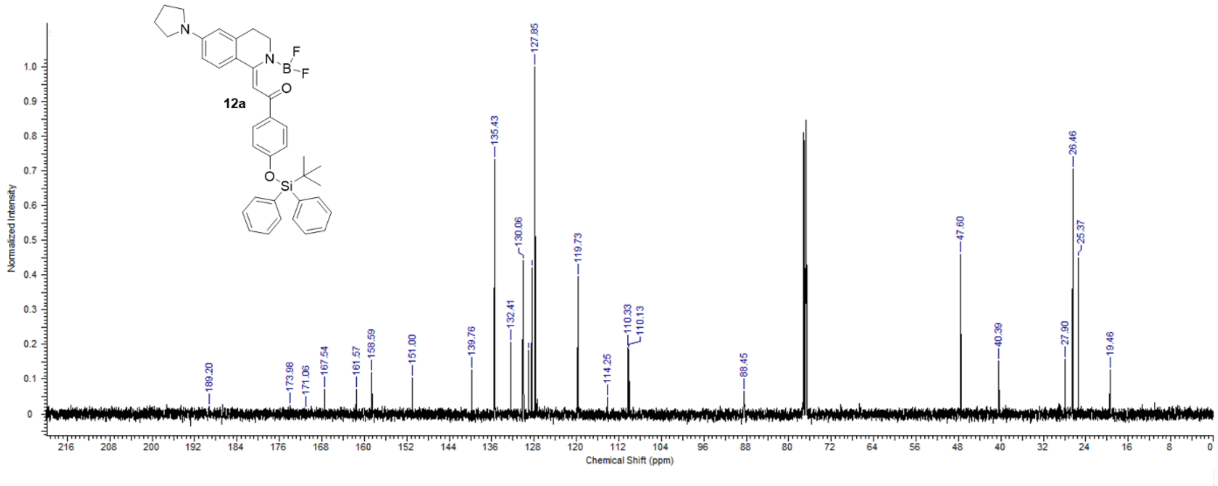

(Z)-allyl (4-(2-(2-(difluoroboranyl)-6-(pyrrolidin-1-yl)-3,4-dihydroisoquinolin-1(2H)-ylidene)acetyl)phenyl)

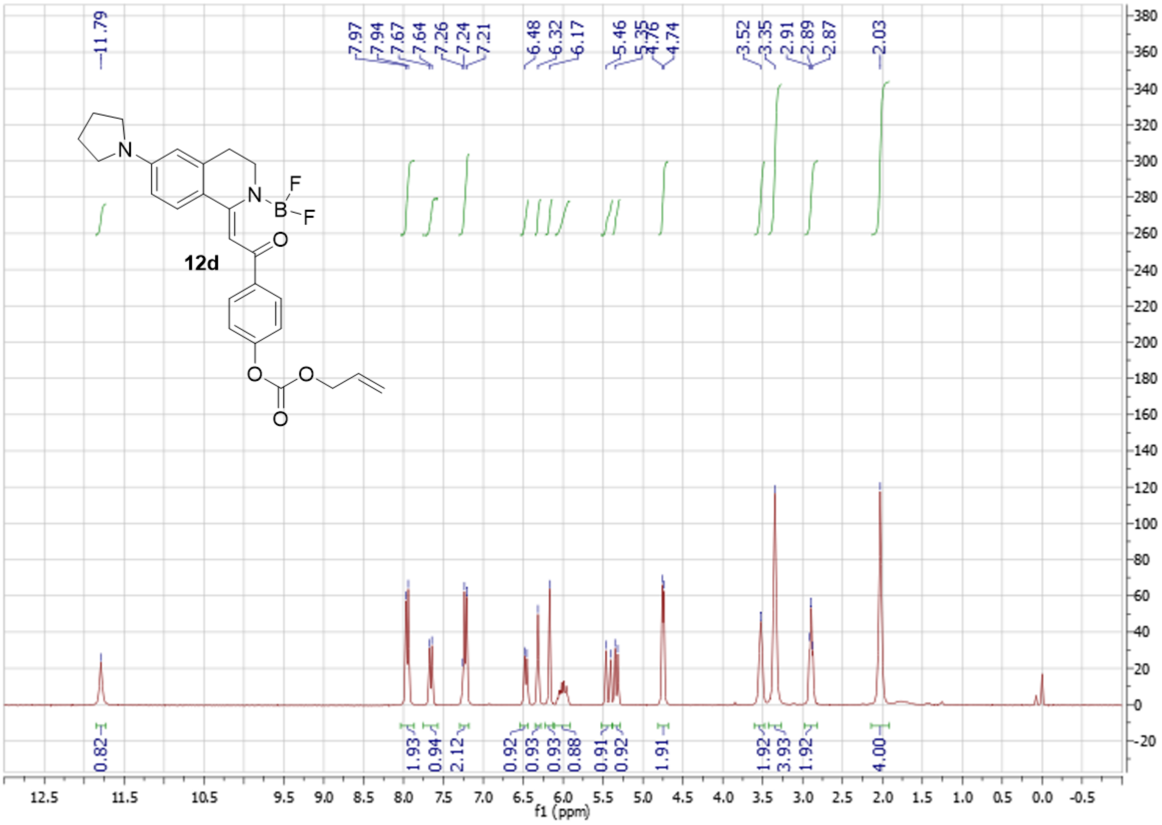

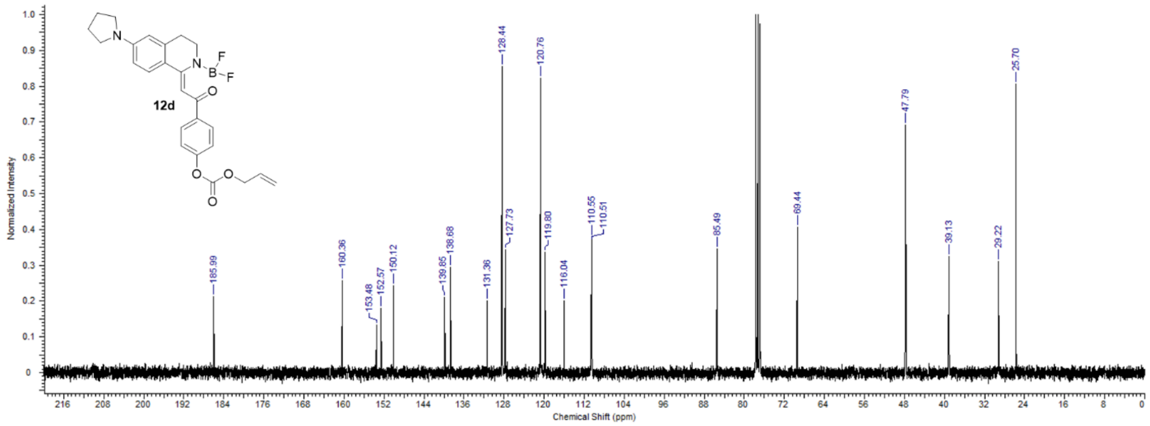

(Z)-2-(2-(difluoroboranyl)-6-(pyrrolidin-1-yl)-3,4-dihydroisoquinolin-1(2H)-ylidene)-1-(4-hydroxyphenyl)ethan-1-one (12c)

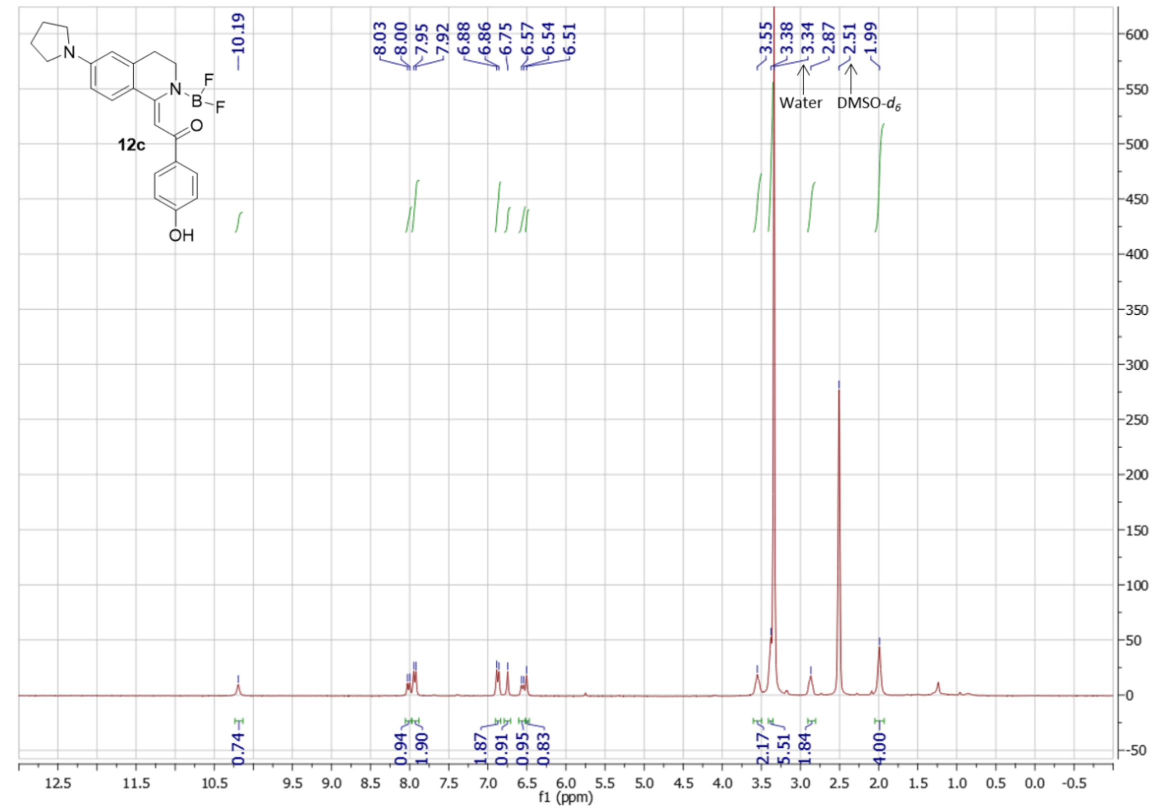

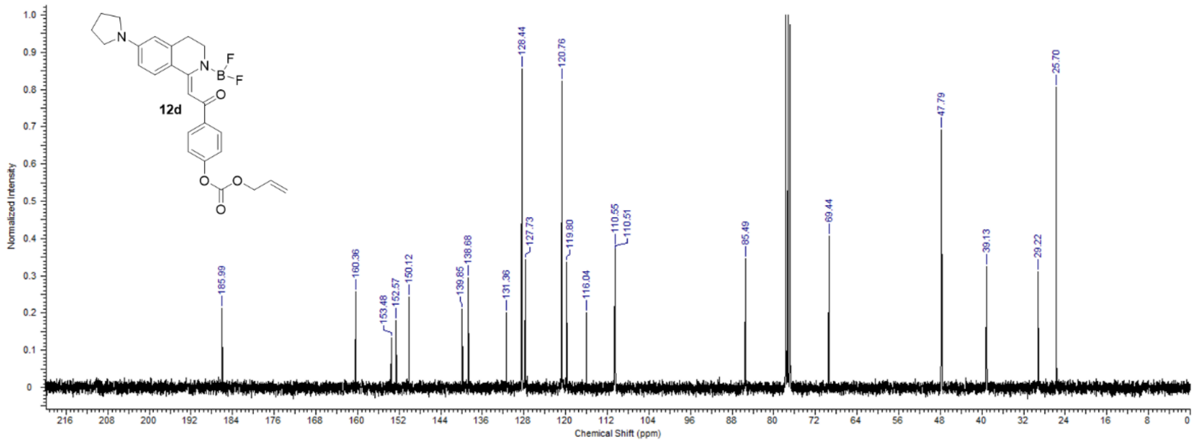

References

1. Langlois, M.; Bremont, B.; Shen, S.; Poncet, A.; Andrieux, J.; Sicsic, S.; Serraz, I.; Mathe-Allainmat, M.; Renard, P.; Delagrange, P. Design and Synthesis of New Naphthalenic Derivatives as Ligands for 2-[125I]Iodomelatonin Binding Sites. *J. Med. Chem.* **1995**, *38*, 2050–2060.
2. Sóvári, D.; Kormos, A.; Demeter, O.; Dancsó, A.; Keserű, G.M.; Milen, M.; Ábrányi-Balogh, P. Synthesis and fluorescent properties of boroisoquinolines, a new family of fluorophores. *RSC Adv.* **2018**, *8*, 38598–38605.
3. Guggilapu, S.D.; Prajapti, S.K.; Babu, B.N. An efficient one-pot oxidative esterification of aldehydes to carboxylic esters using B(C6F5)3–TBHP. *Tetrahedron Lett.* **2015**, *56*, 889–892.
4. Gibson, H.W.; Lee, S.H.; Engen, P.T.; Lecavalier, P.; Sze, J.; Shen, Y.X.; Bheda, M. New triarylmethyl derivatives: “blocking groups” for rotaxanes and polyrotaxanes. *J. Org. Chem.* **1993**, *58*, 3748–3756.
